# Rituximab for the Treatment of IgG4-Related Tubulointerstitial Nephritis: Case Report and Review of the Literature

**DOI:** 10.1097/MD.0000000000001366

**Published:** 2015-08-14

**Authors:** Blaithin A. McMahon, Tessa Novick, Paul J. Scheel, Serena Bagnasco, Mohamed G. Atta

**Affiliations:** From the Division of Nephrology (BAM, PJS, MGA); Osler Medical Residency Program (TN); and Division of Pathology, Johns Hopkins University School of Medicine, Baltimore, Maryland (SB).

## Abstract

Immunoglobulin type gamma 4 (IgG4)-related disease is a relatively newly described clinical entity characterized by a distinctive histopathological appearance, increased numbers of IgG4 positive plasma cells and often, but not always, elevated serum IgG4 concentrations. The most common renal manifestation of IgG4-related disease is tubulointerstitial nephritis marked with proteinuria, hematuria, decreased kidney function, hypocomplementemia, and radiologic abnormalities. Renal biopsy characteristics include dense lymphoplasmacytic tubulointerstitial nephritis that stains for IgG4, storiform fibrosis, and immune complex deposition in the interstitium and along tubule basement membranes. Treatment traditionally consists of prolonged glucocorticoids but cases refractory to glucocorticoids have been reported.

We report a case of a 58-year-old Caucasian man who presented with fatigue, 50 pound weight loss, dyspnea, lymphadenopathy, and nephromegaly. The patient was first misdiagnosed as chronic interstitial nephritis secondary to renal sarcoid and was treated with repeated doses of prednisone. On his third relapse, he underwent a repeat renal biopsy and a diagnosis of IgG4-tubulointerstitial nephritis was confirmed. He was refractory to treatment with prednisone. The patient received Rituximab and had prompt sustained improvement in renal function. At 1 year post Rituximab treatment, his serum creatinine remains at baseline and imaging study revealed reduction in his kidney size.

This is the first case report using Rituximab as a steroid sparing option for refractory IgG4-tubulointerstitial nephritis. More information is needed on the long-term effects of using of B-cell depleting agents for glucocorticoid resistant IgG4-tubulointerstitial nephritis.

## INTRODUCTION

Immunoglobulin type gamma 4-related disease (IgG4-RD) is a newly described proinflammatory disorder defined by the combined presence of the characteristic histopathological appearance (lymphoplasmacytic infiltration, storiform fibrosis, and obliterative phlebitis), increased numbers of IgG4-positive plasma cells, and often, but not always, elevated serum IgG4 concentrations.^1^ Renal involvement predominantly consists of tubulointerstitial nephritis (TIN), and ongoing tubulointerstitial inflammation and fibrosis are thought to cause progressive decline in renal function.^2^

The optimal treatment for IgG4-RD is unknown, and is largely based on retrospective case series.^3^ The mainstay of treatment is glucocorticoid therapy, which tends to induce rapid disease remission.^4,5^ Nevertheless, relapses are common, and necessitate prolonged glucocorticoid courses during which additional organ involvement often develops.^3–5^ Immunomodulatory agents have been used as treatment alternatives in cases of relapsing autoimmune pancreatitis (AIP) and IgG4-related sclerosing cholangitis.^4,6^ In particular, the B-cell depleting agent Rituximab has been documented as a successful glucocorticoid sparing option in several case series (Table 1) and in a recent prospective single-arm trial.^3,4,6,7^ There are limited data for Rituximab use in IgG4-TIN.

**TABLE 1:**
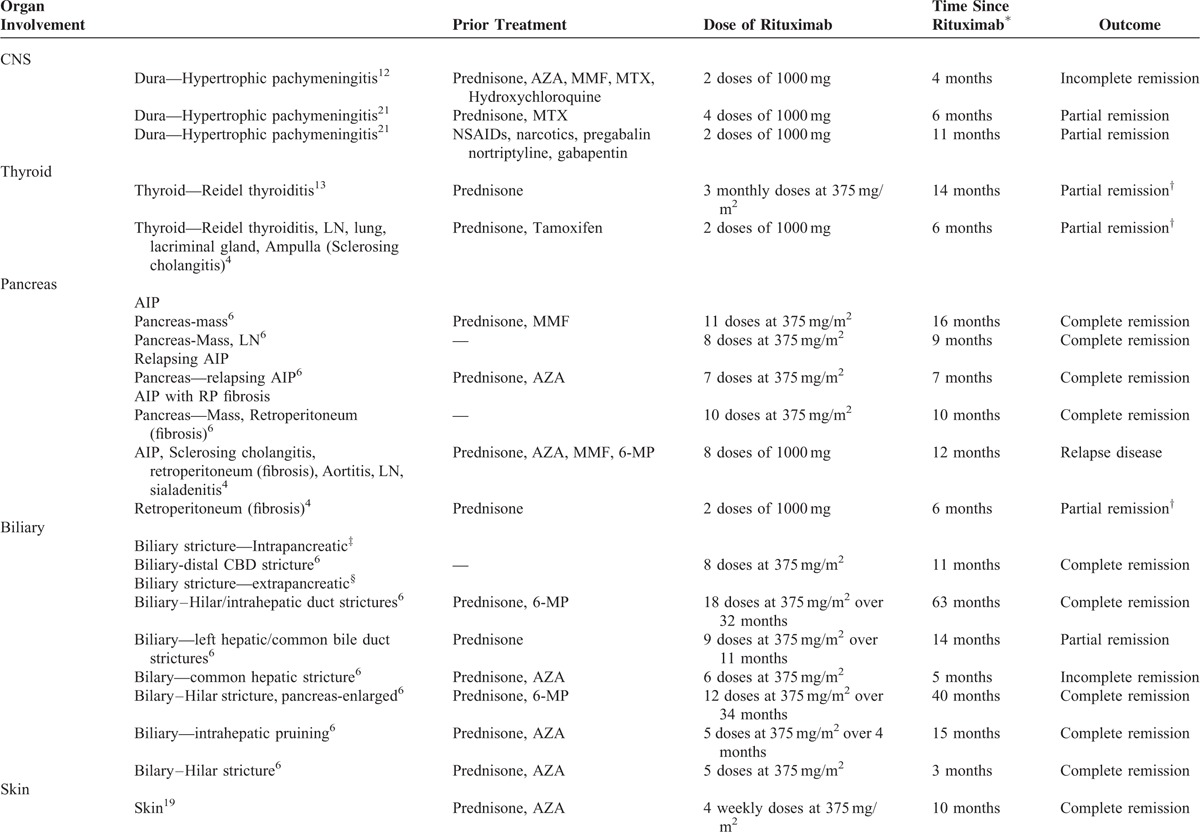
Profiles of IgG4-Related Disease Cases Treated With Rituximab

**TABLE 1 (Continued):**
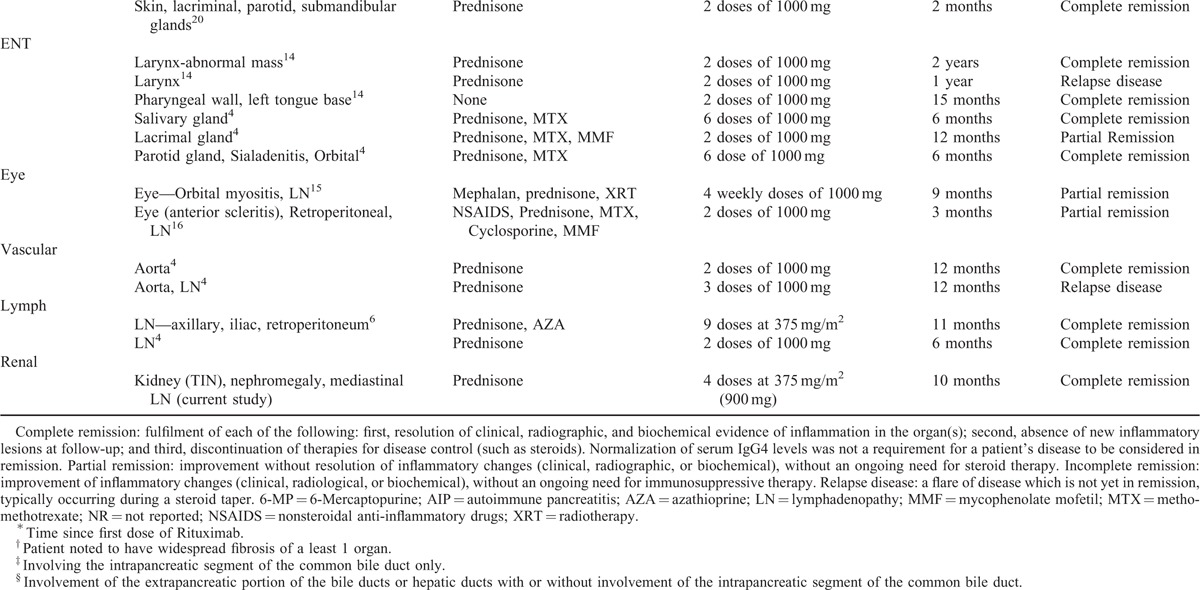
Profiles of IgG4-Related Disease Cases Treated With Rituximab

Herein, we report a patient with a classic clinical presentation for IgG4-TIN, who progressed despite prolonged treatment with glucocorticoids. We review the histopathological features of his kidney biopsy. We also describe the patient's dramatic clinical improvement 1 year after the administration of Rituximab despite the severe interstitial fibrosis and tubular atrophy seen on his renal biopsy. A brief summary of Rituximab use in nonrenal IgG4-related disease is provided.

## METHODS

Informed consent was obtained.

### Case Report

A 58-year-old Caucasian man presented with fatigue, 50 pound weight loss, and dyspnea. Significant medical history included hypertension, diabetes, bipolar disorder, monoclonal gammopathy of undetermined significance, and remote stage 2 adenocarcinoma of the sigmoid colon treated surgically without chemotherapy or radiation (6 years prior). He initially presented with these symptoms to his oncologist during an annual follow-up. His physical examination was notable for stage 1 hypertension, BMI 30, relative euvolemia with no chest, cardiac, or abdominal abnormalities. His kidneys were not ballotable. He had no palpable lymphadenopathy and no skin lesions. Laboratory work revealed a creatinine of 2.1 mg/dL, up from 0.9 mg/dL 7 months prior, corresponding to a decline in his estimated glomerular filtration rate (eGFR) from 66 mL/min/1.73 m^2^ to 33 mL/min/1.73 m^2^. Urinalysis showed 1 + protein was negative for white and red blood cells, and without active sediment. He had a urinary protein to creatinine ratio of 373 mg/g. Initial work-up was notable for antinuclear antibody titers of 1:640 (homogeneous pattern), rheumatoid factor of 120, C3 of 105 mg/dL, C4 of <5 mg/dL, and erythrocyte sedimentation rate of 134 mm/hr. His complete blood count and complete metabolic panels were unremarkable. All further inflammatory and infectious studies were negative, including angiotensin-converting enzyme, antinuclear cytoplasmic antibodies, cryoglobulins, anti-RNP/Smith antibodies, serum and urine protein electrophoresis, hepatitis panel, and testing for human immunodeficiency virus.

**FIGURE 1:**
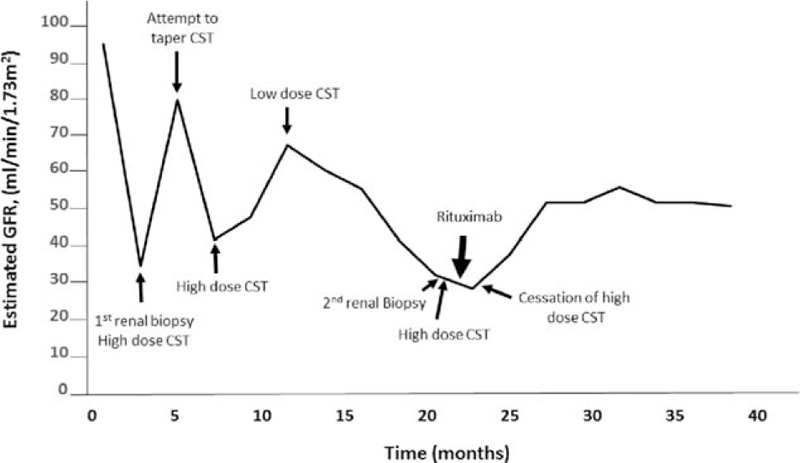
Patient's trend in estimated GFR over a 3-year period. Initially, his decline in renal function was treated with prednisone (60 mg/day). Attempts to taper prednisone resulted in rebound elevations in serum creatinine. After his third relapse in renal function, a repeat renal biopsy confirmed the diagnosis of IgG4-related renal disease. He was treated again with prednisone, but his renal function failed to improve and was subsequently treated with Rituximab (4 weekly doses of 375 mg/m^2^ (900 mg)). Corticosteroids were stopped 9 weeks after the 2nd renal biopsy. One year after treatment with Rituximab, estimated GFR was 50 mL/min/1.73 m^2^ and he was corticosteroid-free. CST =  corticosteroids; GFR = glomerular filtration rate.

**FIGURE 2:**
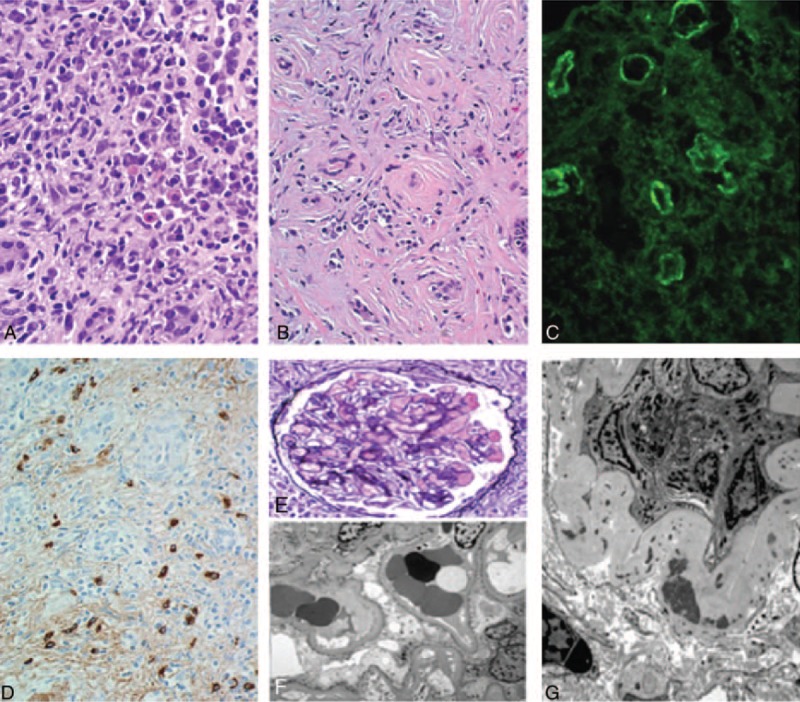
Renal biopsy. A, Plasma cell rich interstitial infiltrate (H&E 600×); B, Storiform pattern of interstitial fibrosis (H&E, 400×); C, Immunofluorescence shows strong IgG peritubular granular staining and faint granular staining in the surrounding interstitium; D, IgG4-positive plasma cells in the interstitium, with faint peritubular and interstitial granular staining (400×); E, Unremarkable glomerulus (PAS-MS, 600×); F, Electron microscopy showing normal glomerular ultrastructural features and absence of electron dense deposits; G, Electron microscopy showing electron dense deposits in the tubular basement membrane.

Computed tomography showed mediastinal lymphadenopathy, and calcified nodules in his lungs and spleen. Both kidneys were enlarged (right kidney measured 17.4 cm × 8.5 cm × 7.5 cm and left kidney measured 16.3 cm × 7.7 cm × 9.5 cm). There was persistent heterogeneity of the renal parenchyma and a soft tissue thickening was noted around the proximal renal collecting system bilaterally. His renal ultrasound also showed bilateral nephromegaly (right kidney 17.4 cm, left kidney 16.3 cm) without lesions or masses. He was referred to a nephrologist and kidney biopsy was performed, which showed chronic interstitial nephritis (not shown). Due to his biopsy findings, mediastinal lymphadenopathy and calcified nodules on imaging a diagnosis of sarcoidosis was suspected, and he was started on prednisone 60 mg with a 2-month taper to a maintenance dose of 10 mg. While consuming high-dose prednisone, the patient was hospitalized for an acute manic episode and elevated blood sugar levels resulting in a change in his oral hypoglycemic drug regimen. His creatinine initially fell to 1.0 mg/dL, but a further attempt to taper prednisone resulted in a rebound elevation in his creatinine again treated with high-dose prednisone that was tapered over 2 months. For 7 months he was maintained on prednisone 10 mg/day and despite this he suffered a third relapse in renal function (with peak creatinine values of 2.2 mg/dL) (Figure 1). As a result of glucocorticoid resistant disease and intolerable treatment side-effects, he was referred to our institution for a second opinion. A repeat kidney biopsy was performed; his eGFR at the time of biopsy was 35 mL/min per 1.73 m^2^. Light microscopy (Figure 2A) showed diffuse, variably dense inflammatory infiltrate in the interstitium. The inflammation did not show granulomatous features. There was extensive tubular loss, interstitial fibrosis, and atrophy (Figure 2B). The tubular atrophy was estimated to involve at least 90% of the cortical and medullary parenchyma. The fibrosis occasionally had a “storiform” pattern (Figure 2B). Immunofluorescence showed strong IgG peritubular granular staining and faint granular staining in the surrounding interstitium (Figure 2C). Immunofluorescence was also notable for fine granular staining of C3, C1q, kappa, and lambda light chains along tubule basement membranes and in the interstitium (not shown). Plasma cellular immunostaining for IgG4 demonstrated predominant IgG4-positive plasma cells (Figure 2D). Glomeruli showed mild expansion of the mesangial matrix, but there were no proliferative, microangiopathic, necrotizing, or cresentic lesions (Figure 2E). Electron microscopy showed normal glomerular ultrastructural features without electron dense deposits (Figure 2F), but electron dense deposits in the tubular basement membrane (Figure 2G).

The presence of plasmacytic-rich inflammatory infiltrate in the interstitium, storiform pattern of interstitial fibrosis, IgG4-positive plasma cells, and immune complex deposition along tubular basement membrane were diagnostic of IgG4-TIN and IgG4-RD.^8,9,18^ Post biopsy his serum IgG4 level was 139 mg/dL. He was treated with glucocorticoid monotherapy (prednisone 60 mg/day × 4 weeks). Despite this, the patient's creatinine continued to rise to 2.2 mg/dL. Given the patient's glucocorticoid resistance and intolerances, he was treated with Rituximab (4 weekly doses of 375 mg/m^2^ each equating to 900 mg). One year after treatment with Rituximab, his creatinine was 1.5 mg/dL [and has remained free from all immunosuppressive therapy over 1 year (Figure 1)]. Repeat renal imaging 1 year post Rituximab showed a reduction in nephromegaly (right kidney 14.5 cm and left kidney 15.5 cm). The patient's initial symptoms of dyspnea, fatigue, and weight loss had resolved and he had no further manic episodes. In addition, his blood glucose levels had stabilized on low-dose oral glyburide.

## DISCUSSION

IgG4-RD, and in particular IgG4-TIN, typically responds rapidly to glucocorticoid therapy, although an exact treatment protocol has not been established.^1,6,10^ Recently, an international consensus guidance statement on the management and treatment of IgG4-related disease was published summarizing treatment strategies used in different parts of the world and involving 8 medical subspecialties.^10^ In this statement, glucocorticoids are postulated to be the first-line agent for induction of remission in all patients with active, untreated IgG4-RD unless contraindications to such treatment exist. Prednisone 30–40 mg/day was considered a common initial treatment dose. Even with the use of maintenance glucocorticoids, relapses in IgG4-RD patients are common.^1,6,10^ Following relapse disease, there is a consensus in North America and Europe that the combined introduction of glucocorticoid and glucocorticoid-sparing immunosuppressive agents from the start of treatment is appropriate in some patients.^10^ However, practice styles vary significantly across countries outside of North America and Europe with regard to use of a second immunosuppressive agent in addition to glucocorticoids from the start of treatment and may relate to lack of access to glucocorticoid-sparing agents.^10^

Our patient initially achieved disease remission with glucocorticoids; however, the disease resistance seen after his third relapse may have been related to the dense interstitial fibrosis and atrophy seen on repeat renal biopsy. The degree of fibrosis on histopathology is thought to relate to treatment failure, with more fibrosis correlating to glucocorticoid-resistant disease.^6,11^ Steroid-sparing treatments with B-cell depleting agents such as Rituximab have been used successfully in isolated cases and case series of glucocorticoid-resistant extra-renal IgG4-RD (Table 1).^4,6,11–17,19,20^ In a prospective single-arm pilot trial using Rituximab as a single agent for IgG4-RD, 97% of patients (n = 30) achieved disease response that was maintained at 6 months.^7^ Rituximab can be effective in allowing discontinuation of all immunosuppressant's with durable remission in a number of cases of IgG4-RD (Table 1) with limited evidence for its use in IgG4-TIN. Despite the high degree of interstitial fibrosis and up to 90% tubular atrophy in this case, preservation of renal function was seen with Rituximab. This is in contrast to reports demonstrating partial or no efficacy of Rituximab when used with severely fibrosed organs such as the thyroid gland.^4,13^

Nephromegaly is not an unusual finding in IgG4-TIN, and potentially caused by cellular infiltration in the renal interstitium^2,8,9^ by plasma cells. In 1 study, 4 out of 23 patients with biopsy confirmed IgG4-TIN had nephromegaly (defined as >14.5 cm).^8^ Other radiographic abnormalities of IgG4-TIN include irregularly shaped hypoattenuated lesions and pseudotumor masses.^2,8^ In our case, Rituximab appeared to reduce his bilaterally enlarged kidneys to a relatively normal size (right kidney previously 17.4 cm was now 14.5 cm, left kidney previously 16.3 cm and now 15.5 cm). The patients estimated GFR normalized at his baseline GFR of 50 mL/min/1.73 m^2^.

In general, Rituximab is well tolerated with the most common side effects related to infusion reactions (fever, chills, hypotension, and headache). In a small prospective trial of Rituximab use in IgG4-RD, there was 1 report of *Klebsiella* urinary tract infection, and 1 report of cold agglutinin-mediated hemolytic anemia.^7^ In 1 systematic review, Rituximab was associated with increased leukopenia and cardiovascular morbidity.^11^ However, evidence from long-term large-scale data is lacking. Our patient tolerated Rituximab well and did not develop any immediate side-effects. More importantly, he was free from glucocorticoids 9 weeks after his initial diagnosis and more than 1 year after his first dose of Rituximab.

In summary, we describe the first case report of successful treatment of glucocorticoid refractory IgG4-TIN with Rituximab. Rituximab provided the opportunity for our patient to be free from glucocorticoids 9 weeks after his initial diagnosis. Our patient was a select case, he had an intolerance to glucocorticoids given his mania and hyperglycemia, but he also had lack of clinical effect with glucocorticoids after his third relapse with IgG4-renal disease. This provided the bases for use of an alternative immunosuppressive agent such as Rituximab, in the treatment of IgG4-TIN. Despite the significant degree of tubular atrophy and fibrosis seen on initial biopsy, our patient had preserved renal function and remained free of disease progression and dialysis over 1 year post Rituximab treatment. Rituximab afforded the opportunity to treat IgG4 disease without the toxicities observed by glucocorticoids. Despite the successful use of Rituximab in the treatment of refractory IgG4-TIN in this case, it should highlight glucocorticoids remain the first-line treatment for most patients with IgG4 disease and B-cell depleting agents may be considered in those patients with a major contraindication to glucocorticoid therapy or an incomplete response to glucocorticoids as observed in our patient.^10^ A randomized clinical trial is needed to define the optimal use and to determine the appropriate role of B cell depletion in IgG4-RD. More information is needed on the long-term effect of using of B-cell depleting agents for glucocorticoid-resistant IgG4-TIN, and the trajectory of disease in these patients.
